# Cancer-associated fibroblasts promote cisplatin resistance in bladder cancer cells by increasing IGF-1/ERβ/Bcl-2 signalling

**DOI:** 10.1038/s41419-019-1581-6

**Published:** 2019-05-10

**Authors:** Xingbo Long, Wei Xiong, Xiting Zeng, Lin Qi, Yi Cai, Miao Mo, Huichuan Jiang, Bisong Zhu, Zhi Chen, Yuan Li

**Affiliations:** 1 Department of Urology, Xiangya Hospital, Central South University, Changsha, 410008 Hunan China; 2 Department of Urology, Beijing Hospital, National Centre of Gerontology, Graduate School of Peking Union Medical College, 100730 Beijing, China; 3 Department of Ophthalmology, Xiangya Hospital, Central South University, Changsha, 410008 Hunan China

**Keywords:** Cancer microenvironment, Chemotherapy

## Abstract

While cancer-associated fibroblasts (CAFs) in the tumour microenvironment may play important roles in bladder cancer (BCa) progression, their impacts on BCa chemoresistance remain unclear. Using human BCa samples, we found that tumour tissues possessed more CAFs than did adjacent normal tissues. Both the presence of CAFs in the BCa stroma and the expression of ERβ in BCa contribute to chemoresistance, and CAFs and BCa cells interact to affect ERβ expression. In vitro co-culture assays demonstrated that compared with normal bladder cells, BCa cells had a higher capacity to induce the transformation of normal fibroblasts into CAFs. When BCa cells were co-cultured with CAFs, their viability, clone formation ability and chemoresistance were increased, whereas their apoptotic rates were downregulated. Dissection of the mechanism revealed that the recruited CAFs increased IGF-1/ERβ signalling in BCa cells, which then led to the promotion of the expression of the anti-apoptotic gene Bcl-2. Blocking IGF-1/ERβ/Bcl-2 signalling by either an shRNA targeting ERβ or an anti-IGF-1 neutralizing antibody partially reversed the capacity of CAFs to increase BCa chemoresistance. The in vivo data also confirmed that CAFs could increase BCa cell resistance to cisplatin by increasing ERβ/Bcl-2 signalling. The above results showed the important roles of CAFs within the bladder tumour microenvironment, which could enhance BCa chemoresistance.

## Introduction

Bladder cancer (BCa) is one of the most common types of urinary tract malignancy^1^. At initial diagnosis, approximately 30% of patients are diagnosed with muscle-invasive BCa (MIBC)^2^. Chemotherapy is a major treatment choice for MIBC patients, especially for high-risk patients, and this approach can effectively inhibit tumour growth and postpone tumour recurrence^3,4^. Cisplatin serves as the first-line chemotherapy for advanced BCa. Unfortunately, most patients benefit little from chemotherapy due to chemoresistance^3^. Growing evidence indicates that the tumour microenvironment may play crucial roles in inducing the acquisition of chemoresistance in various tumours, and this microenvironment has become a major focus in modelling therapeutic responses^5–7^. Cancer-associated fibroblasts (CAFs), the activated phenotype of fibroblasts within tumours, are the most abundant stromal cells in various tumour microenvironments^8,9^. Recent research has shown that CAFs not only play an important role in tumour growth, angiogenesis and dissemination but also regulate chemoresistance in multiple types of solid tumour cells, including breast, ovarian and lung cancer^10–12^. However, to the best of our knowledge, few studies have discussed the role of CAFs in the acquisition of chemoresistance by BCa.

Epidemiological studies have demonstrated that women have a lower incidence of BCa, yet when they do have the disease, women have higher rates of MIBC and recurrence and a poorer prognosis than do men^13,14^, indicating that oestrogen and oestrogen receptors (ERs) may play critical roles in the initiation and proliferation of BCa through specific receptor-induced signalling pathways. ER alpha (ERα) and ER beta (ERβ) are two major types of ERs that mediate the effects of oestrogen in various tissues^15^. Compared with ERα, ERβ has been proven to be more predominant in human bladder and BCa tissues^16,17^. Moreover, an increasing number of clinical studies has demonstrated that ERβ is a biomarker correlated with poor clinical outcomes in BCa patients^17–20^. In addition, several in vivo and in vitro studies have suggested that ERβ signalling pathways are involved in progression, invasion and anti-apoptotic events in BCa cells^21,22^. However, the relationship between the ERβ signalling pathway and BCa chemoresistance and the potential mechanisms involved remain unclear. Herein, our study aimed to investigate whether CAFs regulate the sensitivity of BCa cells to chemotherapy and whether ERβ is involved in the effect of CAFs on BCa cells.

## Results

### CAFs and ERβ were correlated with cisplatin-based chemosensitivity and prognosis in MIBC patients

The clinicopathological characteristics of 28 MIBC patients who received cisplatin-based neo-adjuvant chemotherapy (NAC) are described in Supplementary Table S1. At the time of radical cystectomy (RC) after NAC, 7 (25.0%) patients were identified as having a pathologic complete response (pCR, ypT0N0; ypT depicts the pathologic stage after neo-adjuvant treatment), 12 (42.9%) patients were identified as having a pathologic partial response (pPR, ypT0N0 < ypT ≤ ypT1N0, including ypT1/Tis/Ta), and 9 (32.1%) patients were identified as having a pathologic non-response (pNR, ypT ≥ ypT2).

**Fig. 1:**
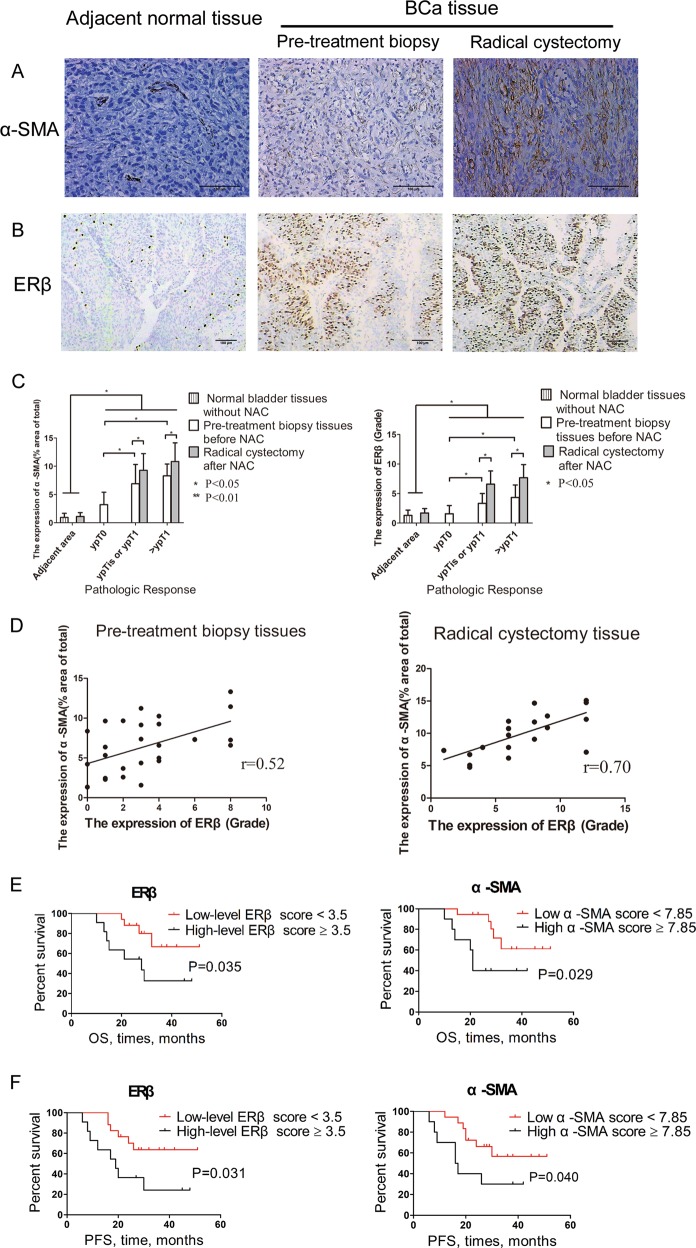
The clinicopathological data of 28 MIBC patients who received cisplatin-based NAC. **a**, **b** The expression of α-SMA (**a**) and ERβ (**b**) in adjacent normal bladder, pretreatment biopsy and resected RC samples (α-SMA ×200, ERβ ×100). **c** α-SMA (left) and ERβ (right) scores in the adjacent normal bladder with or without NAC treatment and in the pretreatment biopsy and resected RC specimens with different pathological outcomes. **d** The correlation of α-SMA expression in the cancer-associated stroma and BCa ERβ expression in the pretreatment biopsy tissues (left) and RC (right) tissues. **e**, **f** Kaplan–Meier survival curves of OS (**e**) and PFS (**f**) in the 28 patients. The results indicated that α-SMA and ERβ expression was related to OS and PFS. Data are presented as the mean ± SD. *P < 0.05

Expression of α-smooth muscle actin (α-SMA) and ERβ in adjacent normal bladder tissues, pretreatment biopsy samples and excised RC specimens were quantified using the methods described in the Supplementary Materials and Methods. The expression of α-SMA, a CAF-specific marker, was found mostly in myofibroblasts, with lower expression in vascular pericytes^23,24^. BCa cells were negative for α-SMA staining (Fig. 1a). ERβ expression was found mostly in tumour cells (Fig. 1b).

Both the α-SMA and ERβ scores were much higher in BCa tissues than in adjacent normal bladder tissues, whereas there was no difference between adjacent normal bladder tissues with and without NAC (Fig. 1c). In the pCR group, the α-SMA and ERβ scores in the pretreatment biopsy tissues were much lower than those in the pPR and pNR groups. In the pPR and pNR groups, the α-SMA and ERβ scores were significantly higher in the RC tissues than in the pretreatment biopsy tissues (Fig. 1c). Moreover, we found that in the pretreatment biopsy tissues, α-SMA expression in the cancer-associated stroma was correlated with ERβ expression in BCa tissues (Fig. 1d, Pearson correlation coefficient = 0.52, *P* < 0.01), and this correlation was much stronger in the RC tissues after NAC treatment (Fig. 1d, Pearson correlation coefficient = 0.70, *P* < 0.001). Univariate analysis showed that a higher cT stage (>cT2) and higher α-SMA and ERβ scores were predictors of a pathologic non-response (Supplementary Table S2). Predictors likely to be associated with a pathologic non-response (*P* < 0.1 in the univariate analysis) were selected in the multivariable analysis; higher cT stage (>cT2) and a higher ERβ score were independent predictors of a pathologic non-response.

To determine if the expression of α-SMA and ERβ in the pretreatment biopsy tissues was associated with the prognosis of MIBC patients receiving cisplatin-based NAC, we analysed overall survival (OS) and progression-free survival (PFS) with the log-rank test and Kaplan–Meier survival curves (Fig. 1e, f). The results showed that both OS and PFS were significantly lower in patients with high levels of α-SMA and ERβ expression than in patients with low levels of α-SMA and ERβ expression.

### Cisplatin accelerated the transition from NFs to CAFs

**Fig. 2:**
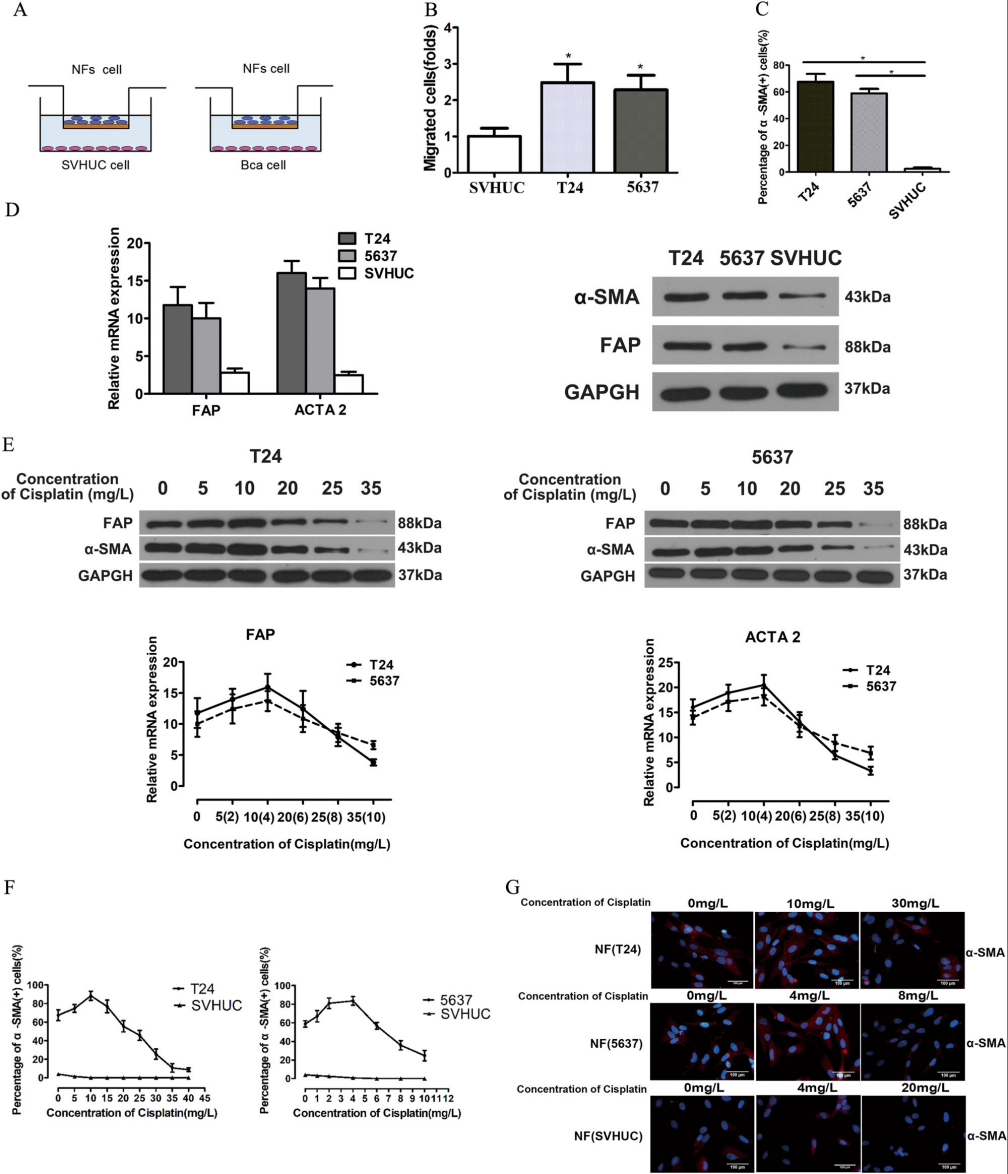
BCa cells can better promote the transformation of NFs into CAFs than non-malignant bladder cells, and appropriate concentrations of cisplatin can accelerate this process. **a** Cartoon showing the transwell co-culture system: CM from BCa cells or SVHUCs was plated into the lower chambers of the transwells, and 1 × 10^5^ NFs cells were plated onto the upper chambers. When implementing fibroblast recruitment assays, a polycarbonate membrane with a 5-μm pore size was inserted between the two chambers. For the noncontact co-culture system, a polycarbonate membrane with a 0.4-μm pore size was used. **b** After 8 h of incubation, the fibroblasts that migrated into the lower chambers were collected and counted. **c** The α-SMA immunofluorescence staining assay was implemented on all fibroblasts in the noncontact co-culture system after 8 h of incubation, and the percentage of α-SMA(+) fibroblasts was counted. **d** Western blot and quantitative real-time PCR (qRT-PCR) results showed the expression of the CAF-specific markers ACTA2 (α-SMA) and FAP in fibroblasts in the noncontact co-culture system after 8 h of incubation. **e** Different concentrations of cisplatin were added to the noncontact co-culture system. After 8 h of incubation, the protein and mRNA levels of ACTA2 and FAP in fibroblasts were analysed. **f**, **g** Different concentrations of cisplatin were added to the noncontact co-culture system. After 8 h of incubation, the percentage of α-SMA(+) fibroblasts was counted in the upper chambers using immunofluorescence. The first column of Fig. 2g (no cisplatin added) is also the experimental condition of Fig. 2c. In the qRT-PCR analysis, we used the β-actin gene as the normalization control. Data are presented as the mean ± SD. *P < 0.05

To compare the capacities of BCa and normal bladder cells to activate normal fibroblasts (NFs) towards CAFs, we applied an in vitro transwell co-culture system. We placed NFs in the upper chambers and then placed BCa cells or normal bladder cells (SV40-transformed human uroepithelial cells, SVHUCs) in the lower chambers (Fig. 2a). When testing the capacity of BCa cells to recruit fibroblasts, we employed a fibroblast recruitment assay. After 8 h of incubation, the number of NFs that migrated through the membranes into the lower chambers was counted and compared with the number of NFs that migrated towards SVHUCs, and we observed that BCa cells had a greater capacity to recruit fibroblasts than did SVHUCs (Fig. 2b). In addition, we employed a transwell noncontact co-culture system to test the capacity of BCa cells to induce the transition of NFs towards CAFs. After 8 h of incubation, an α-SMA immunofluorescence staining assay was implemented on all of the fibroblasts in the upper chambers, and the percentage of α-SMA(+) fibroblasts was determined (Fig. 2c, the first column of Fig. 2g, no cisplatin treatment). In addition, the protein and mRNA levels of ACTA2 (α-SMA) and FAP, two CAF-specific markers, in the fibroblasts in the upper chambers were analysed by western blotting and quantitative real-time PCR (qRT-PCR), respectively (Fig. 2d). The results show that BCa cells had a much higher capacity than the non-malignant SVHUCs to induce NF transformation to CAFs (fibroblasts with high levels of α-SMA and FAP expression) (Fig. 2c, d). Then, we added different concentrations of cisplatin to the conditioned medium (CM) of BCa cells. After 8 h of incubation, we found that at certain concentrations of cisplatin (10 mg/L for the T24 group and 4 mg/L for the 5637 group), the percentage of NFs that transitioned to CAFs was increased. As the concentration of cisplatin further increased, the protein and mRNA levels of ACTA2 and FAP in the fibroblasts and the percentage of α-SMA(+) fibroblasts decreased rapidly (Fig. 2e–g). The above data indicate that compared with non-malignant SVHUCs, BCa cells have an increased capacity to recruit fibroblasts and induce the transformation of NFs to CAFs, and appropriate concentrations of cisplatin can accelerate these processes.

### CAFs enhanced chemoresistance in BCa cells

**Fig. 3:**
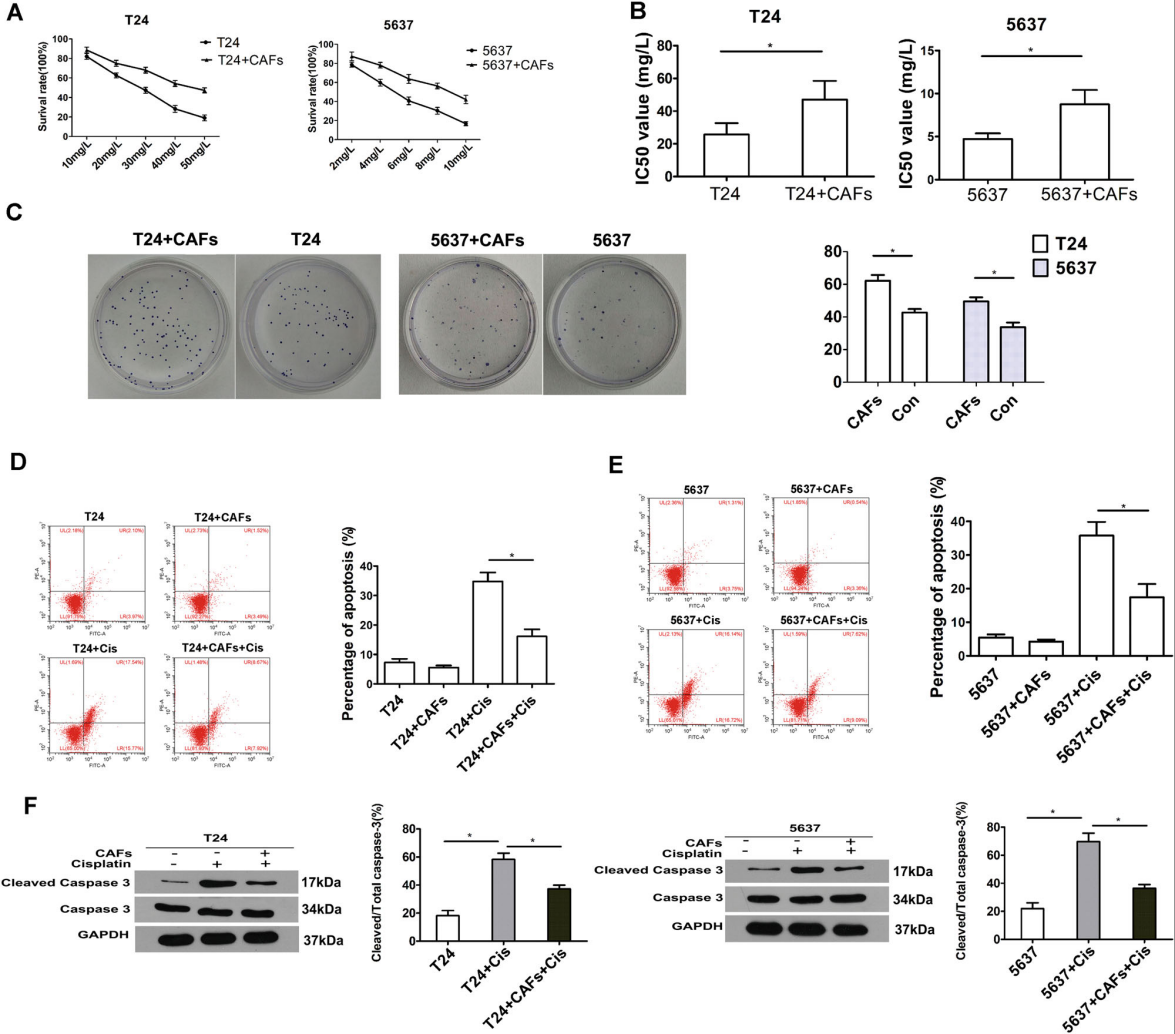
CAFs could reduce BCa cell sensitivity to cisplatin, promote BCa cell proliferation and decrease cisplatin-induced apoptosis. **a** The cell viability with different concentrations of cisplatin as measured by the MTT assay. **b** The IC50 values in each group. **c** Colony formation assay showed that after 2 weeks of culture, compared with the non-co-culture groups, the co-cultured T24 and 5637 cell groups showed increased colony forming ability. All groups were treated with a specific concentration of cisplatin (T24, 25 mg/L and 5637, 6 mg/L). **d**, **e** Apoptotic cells were detected by Annexin V-FITC and PI double staining and analysed by flow cytometry. The result showed that co-culturing reduced cisplatin-induced apoptosis (T24, 25 mg/L and 5637, 6 mg/L respectively). **f** Total and cleaved caspase-3 levels in BCa cells were determined by western blotting for each group. T24 and 5637 cells grown with (+) or without (−) CAF co-culture (24 h) were incubated for 24 h in the presence (+) or absence (−) of 25 mg/L and 6 mg/L cisplatin, respectively. The histogram on the right illustrates the ratio of cleaved caspase-3 and total caspase-3. Data are presented as the mean ± SD. *P < 0.05

Compared with the cells cultured without CAFs, cells (both T24 and 5637 cells) co-cultured with CAFs exhibited dramatically increased survival rates (Fig. 3a). The IC50 results showed that co-culture with CAFs decreased the sensitivity of both T24 and 5637 cells to cisplatin (Fig. 3b). Furthermore, a colony formation assay showed that the proliferation of both cell lines was enhanced after co-culture with CAFs (*P* < 0.05) (Fig. 3c). In addition, flow cytometry revealed that co-culture of either T24 or 5637 cells with CAFs decreased apoptosis of these uroepithelial cell lines (Fig. 3d, e). Finally, we observed that after exposure to cisplatin (T24, 25 mg/L; 5637, 6 mg/L), the levels of cleaved caspase-3, a key mediator of apoptosis, in both T24 and 5637 cells was significantly increased, whereas this value was decreased in the presence of CAFs (Fig. 3f). The above data indicate that co-culture with CAFs could promote proliferation and protect BCa cells from cisplatin-induced apoptosis.

### Silencing ERβ alleviates chemoresistance induced by CAFs in BCa cells

**Fig. 4:**
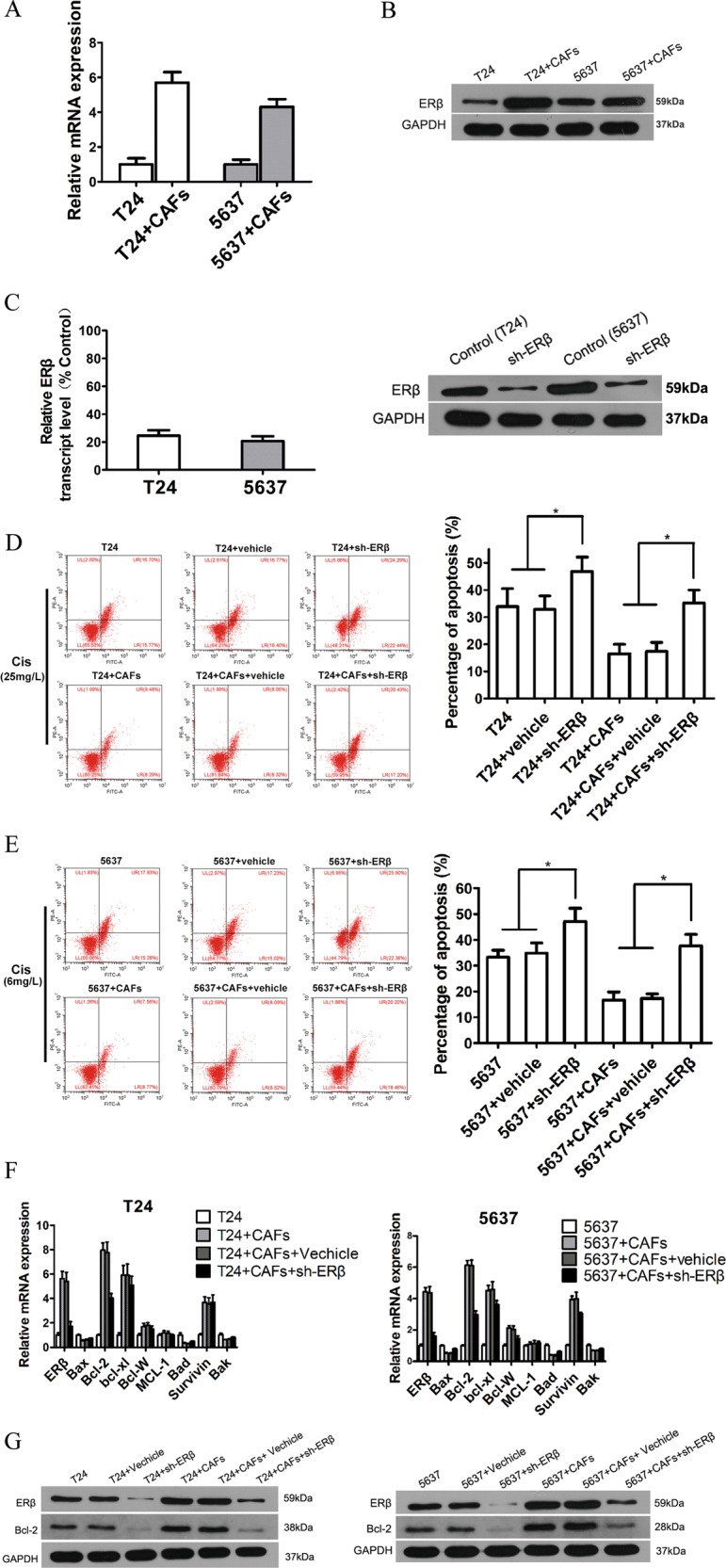
CAFs could decrease BCa apoptosis induced by cisplatin via upregulation of ERβ/Bcl-2 signalling. **a** qRT-PCR results showed increased ERβ expression in BCa cells (T24 and 5637) after co-culture with CAFs. The mean expression value of the control cells (T24, 5637) was defined as 1. **b** Western blotting results showed that ERβ protein expression increased in BCa cells (T24, 5637) after co-culture with CAFs. **c** Validation of the ERβ shRNA knockdown efficiency in T24 and 5637 cells using western blotting (right) and relative mRNA expression (left) levels of ERβ in T24 and 5637 cells at 48 h after transfection. Expression values were shown relative to those of the untreated group (control = 100%) (left). **d**, **e** Knocking down ERβ in BCa cells can reverse the effects of CAFs on BCa cell resistance to apoptosis induced by cisplatin (T24, 25 mg/L; 5637, 6 mg/L). **f** qRT-PCR results showed that Bcl-2 expression levels were significantly increased in both T24 and 5637 cells after co-culture with CAFs and could be partially reversed by ERβ knockdown. The mean expression value of the control cells (T24, 5637) was defined as 1. **g** Western blotting results showed that Bcl-2 expression was increased in T24 and 5637 cells after co-culture with CAFs and decreased after knocking down ERβ. All procedures were conducted after cells were cultured in an appropriate concentration of cisplatin (T24, 25 mg/L; 5637, 6 mg/L). Data are presented as the mean ± SD. *P < 0.05

The mRNA and protein levels of ERβ were increased in BCa cells after co-culture with CAFs (Fig. 4a, b). ERβ was knocked down in BCa cells by ERβ-short hairpin RNA (shRNA), and western blotting (Fig. 4c, right) and qRT-PCR (Fig. 4c, left) analysis verified the ERβ knockdown efficacy 48 h after transfection in both BCa cell types. Suppression of ERβ could reverse CAF-promoted cisplatin resistance in both T24 and 5637 cells (Fig. 4d, e). To further elucidate the mechanisms by which ERβ signalling might contribute to CAF-promoted BCa resistance to cisplatin-induced apoptosis, we screened for the expression of a group of apoptosis-related genes. We found that the expression of the anti-apoptotic gene Bcl-2 was consistently higher in BCa cells after co-culture and could be partially reversed by ERβ knockdown (Fig. 4f). Next, western blotting demonstrated that the protein level of Bcl-2 was selectively increased in the two BCa cell lines after co-culture with CAFs and was decreased after knocking down ERβ (Fig. 4g). The results shown in Fig. 4 proved that CAFs could decrease cisplatin-induced BCa apoptosis via modulation of ERβ/Bcl-2 signalling in BCa cells.

### CAFs regulated ERβ expression via IGF-1/AKT signalling

**Fig. 5:**
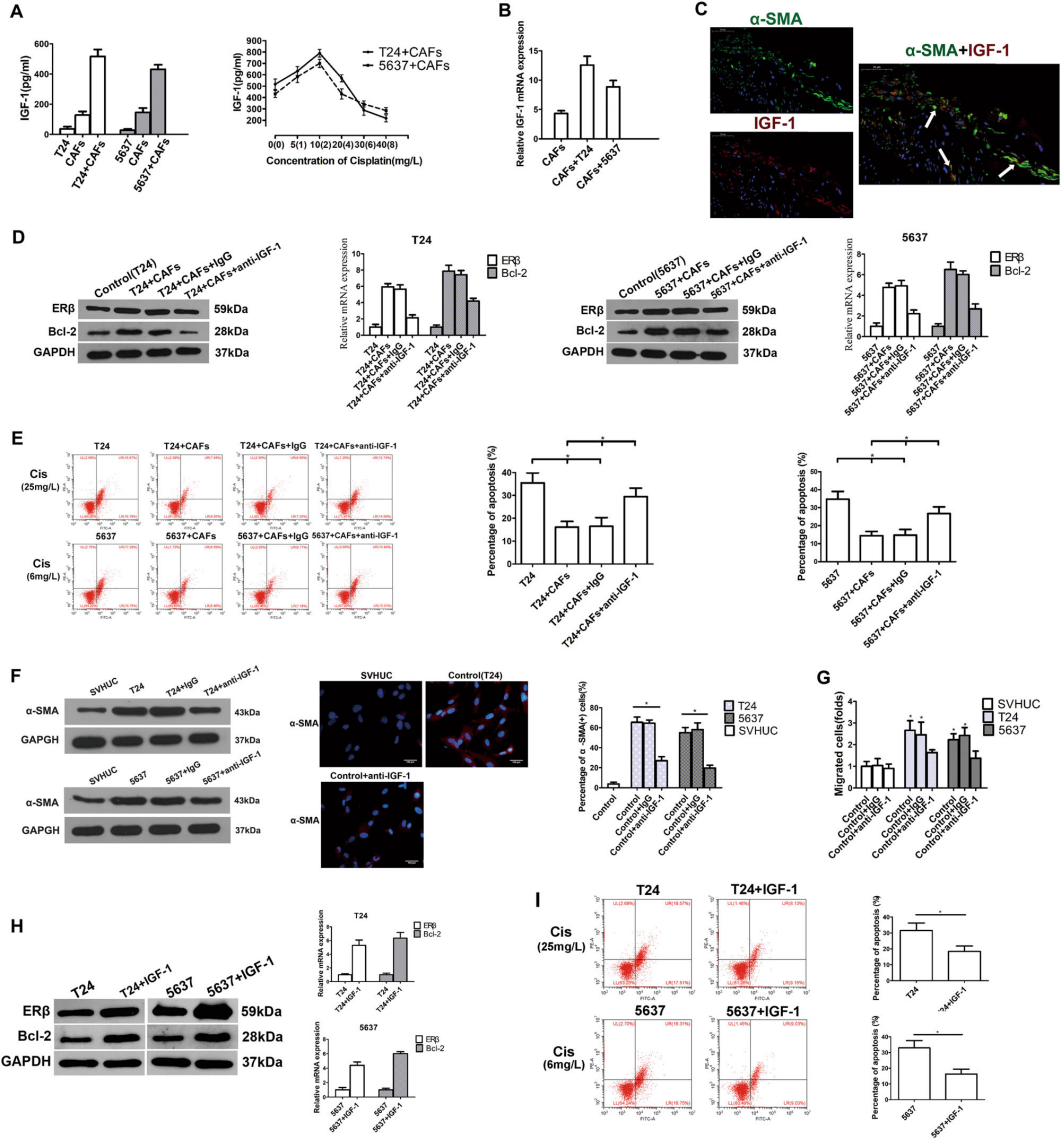
Elucidation of the mechanism by which CAFs increased ERβ expression in BCa cells. **a** IGF-1 levels in the CM from CAFs, BCa cells or co-cultured (CAF+BCa) cells were quantified by ELISA (left panel). IGF-1 levels in the CM of co-cultured cells treated with different concentrations of cisplatin for 48 h were quantified by ELISA (right panel). **b** Assessment of IGF-1 mRNA expression in CAFs by qRT-PCR using the β-actin gene as the normalization control. **c** Immunofluorescence double staining for α-SMA (green) and IGF-1 (red) in pathologic non-response RC tissues (400×). Double-positive areas are indicated (arrows). **d** Western blotting (left panel) and qRT-PCR (right panel) results showed that blocking IGF-1 with an anti-IGF-1 neutralizing antibody can partially reverse CAF-mediated ERβ and Bcl-2 upregulation in BCa cells. The mean expression value of the control cells (T24, 5637) was defined as 1. **e** Blocking IGF-1 can partially reverse the effects of CAFs on BCa cell resistance to apoptosis induced by cisplatin. **f** Western blotting (left) and immunofluorescence staining (right) show that blocking IGF-1 in the co-culture system reversed the capacity of BCa cells to induce NF transformation into CAFs (The expression of α-SMA in the fibroblasts decreased after blocking IGF-1 in the co-culture system). **g** Blocking IGF-1 in the co-culture system reversed the capacity of the BCa to recruit fibroblasts. **h** IGF-1 increased the protein (left panel) and mRNA (right panel) levels of ERβ and Bcl-2 in BCa cells. **i** IGF-1 can increase BCa cell resistance to cisplatin. The procedures in **d**, **e**, **h** and **i** were conducted after the cells were incubated in an appropriate concentration of cisplatin (T24, 25 mg/L; 5637, 6 mg/L). Data are presented as the mean ± SD. *P < 0.05

Previous studies have shown that CAFs may affect chemotherapy resistance through cytokine and chemokine production^25^. A group of cytokines reported to be associated with chemotherapy resistance were screened with their corresponding enzyme-linked immunosorbent assay (ELISA) kits (data not shown). A higher level of insulin-like growth factor 1 (IGF-1), a well-known autocrine and paracrine inducer of CAF activation^9^, was detected in the co-cultured CM than in the CM from cells grown without co-culture (Fig. 5a, left). In the co-culture CM, the level of IGF-1 was even higher in the presence of an appropriate concentration of cisplatin (Fig. 5a, right). qRT-PCR also confirmed that IGF-1 mRNA transcripts were most abundant in CAFs after co-culture (Fig. 5b). Furthermore, in human clinical samples, we found that IGF-1 was abundant in the tumour stroma CAFs in RC tissues from the pNR group (Fig. 5c). Then, 10 µg/mL anti-IGF-1 neutralizing antibody was applied to suppress the effect of IGF-1, and the results indicated that blocking IGF-1 could partially reverse CAF-mediated upregulation of ERβ and Bcl-2 in BCa cells (Fig. 5d) and CAF-mediated promotion of cisplatin resistance in BCa (Fig. 5e). Importantly, blocking IGF-1 in the co-culture system also reversed the capacity of BCa cells to induce the transformation of NFs to CAFs (Fig. 5f) and recruit fibroblasts (Fig. 5g). Next, we added 100 ng/mL IGF-1 to the CM of BCa cells to observe the effects of excess IGF-1. The results suggested increased expression of ERβ and Bcl-2 in the BCa cells (Fig. 5h). Importantly, adding IGF-1 also increased cisplatin resistance of BCa cells (Fig. 5i). These results indicated that the IGF-1/ERβ/Bcl-2 signalling axis might play vital roles in mediating CAF-induced cisplatin resistance in BCa cells.

**Fig. 6:**
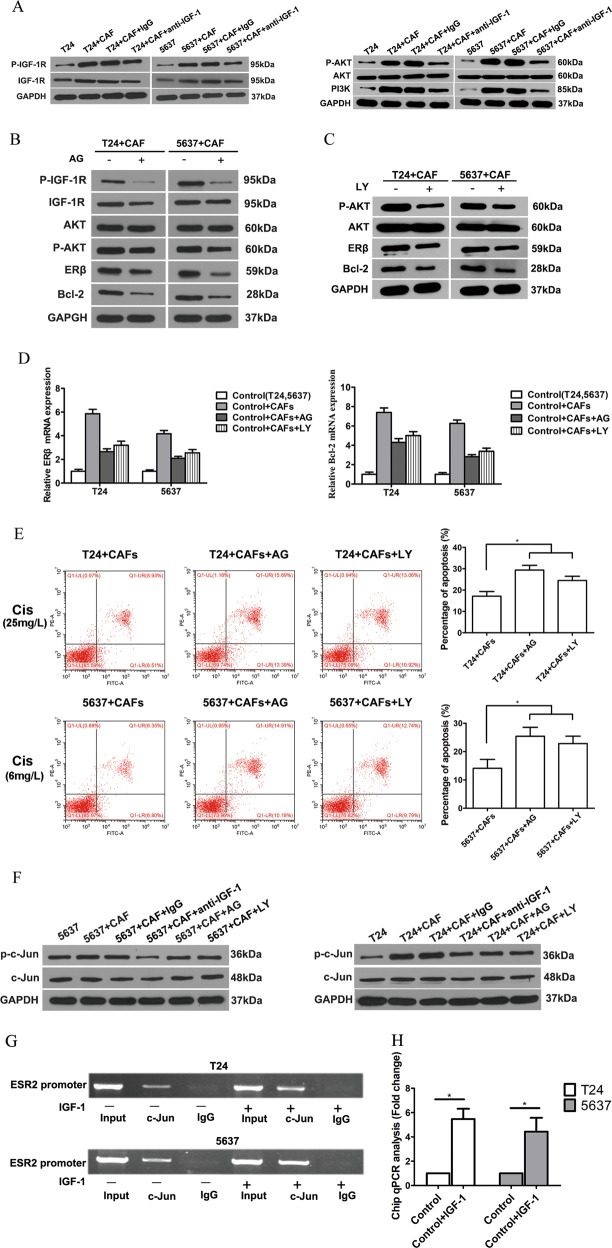
CAFs increased ERβ expression via IGF-1/AKT/c-Jun signalling. **a** The expression of IGF-1R and PI3K and the phosphorylation of the IGF-1R and AKT proteins were increased after co-culture, but these effects were reversed by blocking IGF-1. **b** Western blotting showed that adding the IGF-1R inhibitor AG (1 μM) to the co-culture system could partially reverse CAF-mediated increases in the protein expression of ERβ and Bcl-2 and the phosphorylation of IGF-1R and AKT. **c** Western blotting showed that ERβ and Bcl-2 expression and AKT phosphorylation were decreased when the cells were pretreated with the AKT inhibitor LY (10 μM) in the co-culture system. **d** qRT-PCR results showed changes in ERβ and Bcl-2 mRNA expression in BCa cells after co-culture upon pretreatment with AG (1 μM) and LY (10 μM) for 1 h before co-culture. The mean expression value of the control cells (T24, 5637) was defined as 1.0. **e** Both AG and LY can partially reverse the effects of CAFs on BCa cell resistance to cisplatin-induced apoptosis. **f** Phosphorylation of c-Jun was detected by western blotting in each group. **g** The cells were treated with or without IGF-1 for 8 h. Then, the cells were harvested and subjected to chromatin ChIP with anti-c-Jun or control IgG, followed by qRT-PCR. **h** ChIP products were measured by real-time PCR. The expression value in the control group was defined as 1. All procedures were conducted after cells were cultured in an appropriate concentration of cisplatin (T24, 25 mg/L; 5637, 6 mg/L). Data are presented as the mean ± SD. *P < 0.05

To further study the signals downstream of IGF-1 that are involved in the upregulation of ERβ expression, we investigated the phosphorylation of several molecules, including IGF-1 receptor (IGF-1R) and its downstream signalling protein AKT. The expression of IGF-1R and the phosphorylation of IGF-1R and AKT were increased dramatically in the co-culture system and decreased by blocking IGF-1. However, co-culture did not influence the levels of total AKT protein (Fig. 6a).

To determine whether activation of IGF-1R and AKT by IGF-1 was required for IGF-1-mediated stimulation of ERβ expression in BCa, we used the specific IGF-1R inhibitor AG1024 (AG; Millipore, Boston, MA, USA) and the AKT inhibitor LY294002 (LY; CST, Boston, MA, USA) for further study. We found that AG could partially reverse CAF-mediated activation of the AKT signalling pathways (Fig. 6b). In addition, treatment with both AG and LY could markedly reduce ERβ and Bcl-2 protein (Fig. 6b, c) and mRNA (Fig. 6d) expression after 48 h of co-culture. In addition, flow cytometry showed that both AG and LY could reverse CAF-mediated cisplatin resistance in T24 and 5637 cells (Fig. 3e). Together, these findings indicate that IGF-1 increased ERβ expression via the IGF-1/IGF-1R/AKT signalling axis.

### IGF-1/AKT signalling activated c-Jun phosphorylation and promoted ESR2 gene transcription

To further investigate how AKT signalling increases ERβ expression, we mapped the human ESR2 promoter region, which was found to contain multiple recognition motifs for sequence-specific transcription factors, including c-Jun^26,27^. In addition, previous findings showed that in rat tumour Leydig cells, IGF-1 had the ability to activate c-Jun^28^. Figure 6f shows that phosphorylation of c-Jun proteins was increased dramatically in the co-culture system and decreased by blocking IGF-1, IGF-1R and AKT signalling. Then, a chromatin immunoprecipitation (ChIP) assay was applied to verify the extent of c-Jun binding to the ESR2 promoter in BCa cells. Cells were treated with or without IGF-1 for 8 h, and ChIP with anti-c-Jun antibodies followed by ESR2 promoter PCR was then performed. The results show a significant increase in c-Jun binding after treated with IGF-1 (Fig. 6g, h). All of these results indicate that CAFs increased ERβ expression via IGF-1/AKT/c-Jun signalling.

### CAFs reduced BCa sensitivity to cisplatin in an animal model

**Fig. 7:**
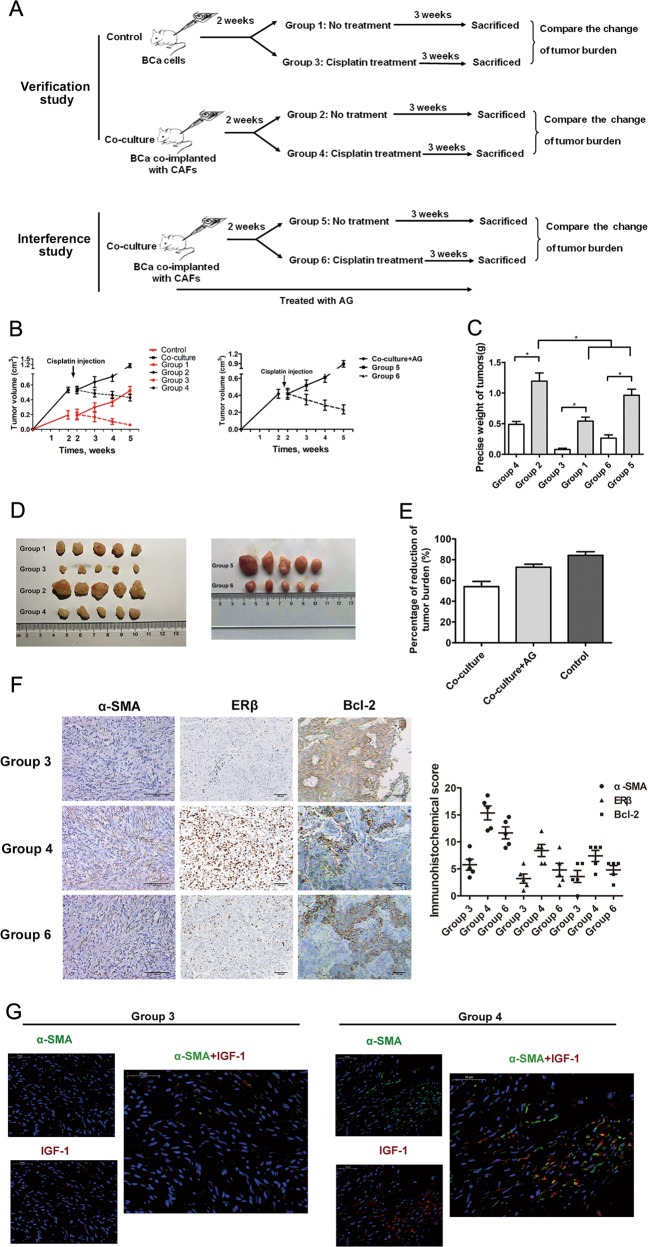
CAFs reduced BCa sensitivity to cisplatin by stimulating IGF-1/ERβ/Bcl-2 signalling in vivo. **a** Grouping and intervention schemes. **b** Changes in the tumour volumes of each group in the verification and intervention study. Tumour volumes were calculated as described in the Materials and methods section. Arrows represent the start of cisplatin treatment, *n* = 5 per group. The mice were sacrificed after completion of the treatments. **c**, **d** Quantification of the tumour sizes in each group in the verification and intervention studies after mice were sacrificed. **e** The percent reduction of tumour burden was calculated to evaluate the effect of cisplatin treatment as follows: percent reduction of tumour burden = (no treatment group – treatment group)/ no treatment group × 100%. **f** IHC staining for α-SMA, ERβ and Bcl-2 in xenograft bladder tumour tissues (α-SMA, 200×; ERβ and Bcl-2, 100×) in each group. **g** Double fluorescence staining of α-SMA (green) and IGF-1 (red) in xenograft bladder tumour tissues (400×). Data are presented as the mean ± SD. *P < 0.05

To confirm these results in an in vivo mouse model, verification and interference studies were performed. The grouping and intervention schemes were as described in the Materials and methods shown in Fig. 7a. In the verification study, we subcutaneously xenografted nude mice with T24 cells co-implanted with or without CAFs. In the interference study, we subcutaneously xenografted nude mice with T24 cells co-implanted with CAFs and treated mice with the IGF-1R inhibitor AG. Figure 7b shows the change in tumour volume during the in vivo verification and interference study. After the mice were sacrificed, the tumours were harvested. The results showed that among the untreated groups, mice in group 2 had a larger tumour size than did mice in group 1 (Fig. 7b–d). To evaluate the therapeutic efficacy of cisplatin in the control, co-culture and interference groups, we compared the differences in the tumour burden between groups 1 and 3 (control groups), groups 2 and 4 (co-culture groups) and groups 5 and 6 (interference groups). The results showed that in all the groups, cisplatin significantly reduced the tumour burden (Fig. 7b–e), whereas the percent reduction of tumour burden was significantly greater in the control group than in the co-culture group. Moreover, blocking IGF-1R could reverse CAF-mediated chemotherapy resistance (interference groups) (Fig. 7e). The results from the immunofluorescence double staining and immunohistochemistry (IHC) staining experiments also indicated that the expression of related key factors, including α-SMA, IGF-1, ERβ and Bcl-2, was much higher in group 4 than in group 3. The expression of ERβ and Bcl-2 was much lower in group 6 than in group 4 (Fig. 7f). An immunofluorescence double staining assay further confirmed that IGF-1 expression in the tumour stroma was closely correlated with the density of CAFs (Fig. 7g).

**Fig. 8:**
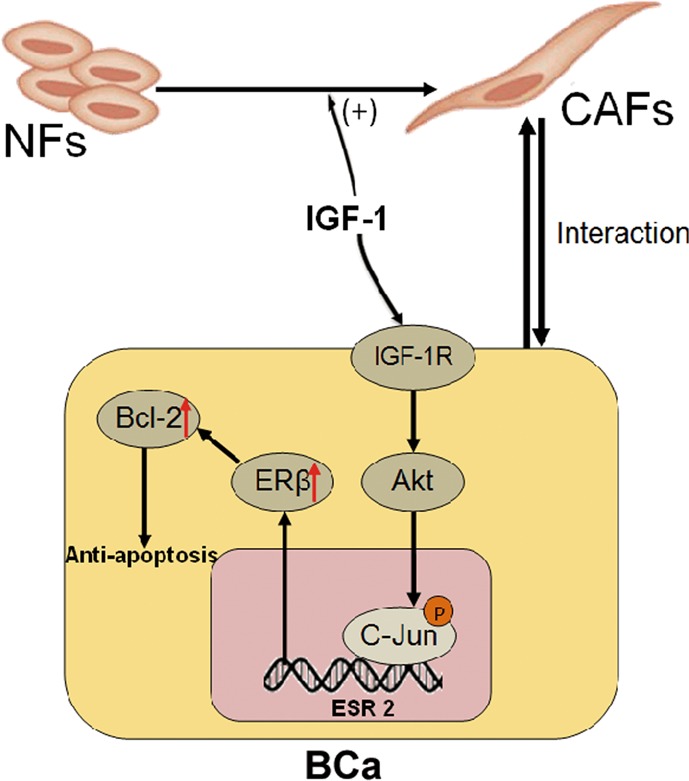
Cartoon illustration summarizing CAF-enhanced cisplatin resistance via stimulating IGF-1/ERβ/Bcl-2 signalling in the tumour microenvironment of BCa. The red arrows indicated an increase in ERβ and Bcl-2 expression

Our data indicate that CAFs in the BCa stroma enhanced cisplatin resistance of BCa by stimulating IGF-1/ERβ/Bcl-2 signalling both in vitro and in vivo (Fig. 8).

## Discussion

Chemoresistance is one major obstacle to improving the chemotherapy outcomes of BCa patients. CAFs, as a major component of the tumour microenvironment, participate in tumour initiation and progression upon activation by expressing proteins such as α-SMA. Herein, our study for the first time explored the nature of BCa resistance to chemotherapy from the perspective of the tumour microenvironment. We found that CAFs in the BCa stroma and the expression of ERβ in BCa were significantly associated with OS in patients treated with chemotherapy.

Previous epidemiological and preclinical studies demonstrated that ERβ signalling may play critical roles in the initiation and progression of BCa^17–22^, whereas few studies have discussed the relationship and potential mechanisms between the ERβ signalling pathway and BCa chemoresistance. We found that ERβ signalling also contributes to BCa chemoresistance, which may be mediated by CAFs. In vitro, we found that co-culture of BCa cells with CAFs significantly increased the expression of ERβ in BCa cells, and we showed that ERβ could be the signalling molecule upstream of Bcl-2 in BCa. Several other studies also demonstrated that Bcl-2 expression was enhanced by ERβ in cardiomyocytes, medulloblastoma cells and BCa cells^21,29,30^. However, one study drew the opposite conclusion that ERβ may reduce Bcl-2 expression in hormone-resistant breast cancer cells^31^.

Unlike ERα, ERβ shows significant oestrogen-independent activities and can be phosphorylated and activated by various signals; in addition, changes in the phosphorylation state of ERβ might alter its ability to activate gene transcription^32^. This finding may explain why ERβ shows different functions in different types of cells and under different conditions. In the present study, we found that ERβ promoted Bcl-2 expression and reduced cancer cell apoptosis.

Previous studies showed that Bcl-2 expression can predict survival in patients receiving synchronous chemoradiotherapy for advanced BCa^33^, which is similar to our results. Upregulation of Bcl-2 protein expression might be one of the mechanisms of cisplatin resistance in BCa cells^34,35^. Consistent with this possibility, an antisense Bcl-2 oligonucleotide was reported to be helpful in reversing cisplatin resistance to chemotherapy in BCa^34^. Here, we proved that CAFs co-cultured with BCa cells could increase Bcl-2 expression and contribute to increased BCa chemoresistance.

CAFs are the most abundant mesenchymal cell type present within the cancer-associated stroma for many cancers, including BCa^35^. CAFs can be derived from different precursor cell types, such as epithelial cells, inflammatory cells, endothelial cells, and bone marrow cells. However, the main source of CAFs is NFs^9^. These precursor cells are activated by a variety of chemokines and cytokines, including IGF-1^9^. In our study, we found that IGF-1 was a vital cytokine in the recruitment and transformation of NFs to CAFs. However, the originating cell of IGF-1 in this context was unclear and required further study. Our preliminary results suggested that both BCa and CAFs contributed to the secretion of IGF-1, but CAFs might be the dominant cell of origin. Cytokines, chemokines and growth factors secreted by fibroblast-like tumour stromal cells can mediate drug resistance in several cancers via induction of gene transcription^5^. In addition, co-culture of BCa with CAFs can enhance the progression of BCa and induce epithelial–mesenchymal transition^36^. In the present study, the in vitro and in vivo data demonstrated that CAFs could also promote cisplatin resistance of BCa.

IGF-1 is a well-known autocrine and paracrine inducer of CAF activation^9^. Elevated expression of IGF-1 and IGF-1R correlates with tumour progression, poor prognosis^37,38^, apoptosis resistance and chemotherapy resistance^39–41^ in several cancer types, including BCa. Inhibiting IGF-1 and IGF-1R signalling has shown promising results in colorectal cancer by blocking the influence of its microenvironment^42^. In our study, blocking IGF-1 in the co-culture system reversed not only CAF-mediated upregulation of ERβ and Bcl-2 and the effects of CAF-mediated cisplatin resistance in BCa but also the capacity of BCa cells to transform NFs into CAFs.

In summary, our results showed that CAFs could promote cisplatin resistance in BCa cells by the IGF-1/ERβ/Bcl-2 signalling pathway. Future studies may facilitate the development of an effective therapeutic strategy to disrupt these newly identified mechanisms in order to reverse resistance to cisplatin in BCa.

## Materials and methods

### Patients

During the median follow-up time of 37.5 (interquartile range, 25.5–47.25) months, 28 patients who had primary resectable MIBC (cT2–4aN0M0) and received at least three cycles of NAC before RC and pelvic lymph node dissection at our institution were retrospectively enrolled in this study. Pretreatment biopsy samples, resected RC samples of the tumour and adjacent normal bladder (3 cm away from the tumour tissues) specimens were collected. Our study included patients with either pure urothelial carcinoma or urothelial carcinoma mixed with squamous and/or glandular differentiation. Patients with cT4b disease and all other variant histologies were excluded from analysis. The study was performed in accordance with the Declaration of Helsinki. Ethics approval was obtained from the ethics committee at Xiangya Hospital, and we obtained written informed consent from all participants in our study. Tumour staging was determined with the 2009 UICC-TNM staging system, and tumour grading was performed according to the World Health Organization (WHO) three-tiered classification^43^. The cisplatin/gemcitabine chemotherapy regimen was administered as follows: gemcitabine 1000 mg/m^2^, once on day 1 and on day 8; cisplatin 70 mg/m^2^, once on day 2. This NAC regimen followed a 21-day cycle and was administered 16 weeks before RC surgery.

To obtain normal bladder specimens unaffected by NAC, adjacent normal bladder specimens from 10 patients with MIBC who underwent RC without NAC were also included in our study.

### Primary human CAF and NF isolation

CAFs and NFs were isolated from human BCa specimens and adjacent normal tissues, respectively, using the method described by Zhuang et al.^44^. Tissues classified as MIBC of histological grade II were used for the isolation of stromal fibroblasts. The tissues were obtained from patients seen at Xiangya Hospital. Dulbecco’s modified Eagle’s medium/F12 medium supplemented with 10% foetal bovine serum (FBS), 100 units/mL penicillin and 100 μg/mL streptomycin was used to collect and culture the isolated cells. Then, a confluent and homogeneous monolayer of stromal fibroblasts was formed after 2–3 passages. The identification and characterization of primary human NFs and CAFs are shown in the Supplementary Materials and Methods and Supplementary Fig. S1.

To prepare CM from BCa cells co-cultured with CAFs, BCa cells were co-cultured with CAFs for 48 h, and the collected medium was centrifuged for 10 min at 3000 rpm to remove cell debris. The fibroblasts used in the experiments were at <10 passages.

### Cell culture

The normal human urothelial cell line SVHUC and the human BCa cell lines T24 and 5637 were obtained from Auragene Bioscience Corporation Inc. Kaighn’s modification of Ham’s F12 medium supplemented with 10% FBS was used to culture the SVHUCs. BCa cells were maintained in RPMI-1640 supplemented with 10% FBS, 100 units/mL penicillin and 100 μg/mL streptomycin. All cells were cultured at 37 °C in a humidified environment containing 5% CO_2_.

### Transwell noncontact co-culture system

We employed a transwell noncontact co-culture system (24 well), with inserts with a 0.4 μm pore size polycarbonate membrane (Corning, #3413, Corning, NY, USA) for all co-culture assays except the fibroblast recruitment assay. A total of 1 × 10^5^ of either CAFs or NFs and BCa cells were plated into the upper and lower chambers of the transwells, respectively. Cells in the upper or lower chambers are unable to migrate through the 0.4 μm pores of the polycarbonate membrane, which creates a noncontact co-culture system.

### Fibroblast recruitment assay

This assay was implemented in a transwell system (24 well) with a polycarbonate membrane containing 5-μm pores (Corning, #3421, Corning, NY, USA). A total of 1 × 10^5^ NFs was plated into the upper chambers of the transwells, and SVHUCs or BCa cells were plated into the lower chambers. We collected and counted the number of fibroblasts that migrated into the lower chamber after 8 h of co-culture.

### MTT assay

BCa cells (with or without co-culture for 24 h) or fibroblasts (CAFs or NFs) in the exponential growth phase were plated in 96-well plates at a density of 3 × 10^3^ cells/well. Next, fresh medium or co-culture medium containing different concentrations of cisplatin was used to replace the original culture medium. Then, after 72 h of culture, 3-(4,5-dimethyl-2-thiazolyl)-2,5-diphenyl-2-H-tetrazolium bromide, thiazolyl blue tetrazolium bromide (MTT) (20 μL/well) was added to each well and incubated with the cells for 2 h, after which an ELISA reader (ELX-800, Bio-Tek) was used to measure the optical density of each well at 570 nm (OD570). GraphPad 5 software (GraphPad Software, CA, USA) was used to calculate the inhibition rate of every concentration and the IC50 (half maximal inhibitory concentration) value of cisplatin on all the tested cells.

### Cell apoptosis assay

After 48 h with or without co-culture, cells from all groups were seeded at a density of 5 × 10^5^ cells per 10 mm dish with fresh or co-culture medium and treated with the indicated concentrations of cisplatin for 24 h. After brief trypsinization, the cells were collected and washed twice in phosphate-buffered saline. After the cells were incubated with 10 μL of propidium iodide (PI) and 5 μL of annexin V-Fluorescein isothiocyanate (V-FITC) at room temperature in the dark for 15 min, they were analysed by flow cytometry (BD, NJ, USA). Cells with a low PI signal and a high annexin V fluorescence signal were regarded as apoptotic cells. The percentages of apoptotic cells were calculated by data obtained from the fluorescence activated cell sorting (FACS) analysis.

### Colony formation assay

For all the groups, 150 cells in 3 mL of medium (either complete medium with the indicated concentration of cisplatin or co-culture CM with the indicated concentration of cisplatin) were seeded in each well of a six-well plate. The plates were incubated at 5% CO_2_, 37 °C for 2 weeks. Then, the cells were gently washed and stained with Giemsa. After the cells were cultured for another 2 weeks, the cloning efficiency was calculated; colonies possessing at least 50 cells were counted.

### Lentivirus packaging and cell transfection

As previously reported, sh-ERβ was constructed in the pLKO.1 lentiviral vector^45^. To prepare the ERβ shRNA lentivirus particles, pLKO.1 sh-ERβ together with packaging and envelope plasmids (psPAX2 and pMD2G, respectively) was transfected into 293T cells. The resulting lentivirus supernatant was collected and stored at −80 °C for later use in the transduction of BCa cells.

### ChIP

First, 1% formaldehyde was added to cross-link the cells, and 10 min later, glycine was used to stop the reaction. To shear genomic DNA from the cross-linked cells, micrococcal nuclease was used to lyse and enzymatically digest the cells. An anti-c-Jun antibody (Abcam) was used for immunoprecipitation, with IgG serving as a negative control. After the protein/DNA complexes were eluted from the beads, they were treated with proteinase K solution for 2.5 h at 65 °C and then analysed by real-time PCR and semiquantitative PCR using primers for the ESR2 promoter. The specific primers used to amplify target sequences from human ERβ promoters are listed in Supplementary Table S3.

### Xenograft mouse model

Six- to 8-week-old female nude mice (BALB/C-nu) were purchased from the animal centre of Xiangya Medical School. All experimental procedures were approved by the Institutional Animal Care and Use Committee of Xiangya Hospital. The total duration of the in vivo experiment was 5 weeks. For the verification study, in the first 2 weeks, the mice were randomly assigned into two groups. In the control group, 10 mice were implanted with 1 × 10^6^ BCa T24 cells only, whereas in the co-culture group, 10 mice were co-implanted with 1 × 10^6^ BCa T24 cells and 1 × 10^5^ CAFs. Three weeks later, the mice were assigned to one of four groups: group 1, control group, no other treatment; group 2, co-culture group, no other treatment; group 3, control group, intravenous injection with cisplatin at 1 mg/kg body weight (Selleck Chemicals, Houston, USA) twice every week; and group 4, co-culture group, intravenous injection of cisplatin as described for group 3. The above-mentioned doses of cisplatin were reported to be effective and safe by previous studies^46^. In the interference study, 10 mice were administered AG (0.2 nM/g) (Millipore) via oral gavage every 3 days throughout the study. During the first 2 weeks, all mice were co-implanted with 1 × 10^6^ BCa T24 cells and 1 × 10^5^ CAFs. Three weeks later, the mice were randomly assigned into two groups: group 5, no other treatment; and group 6, intravenous injection of cisplatin as described for groups 3 and 4.

The grouping and intervention schemes are shown in Fig. 7a. Tumours were measured using a calliper once a week after xenografting until the mice were sacrificed, and the tumour volume was estimated as ab^2^π/6, where *a* represents the largest diameter and *b* represents the largest diameter perpendicular to *a*. When the mice were sacrificed, the precise weights of the tumours were measured, and the tumours were processed for IHC and fluorescence staining.

### Statistical analysis

All data are presented as the mean ± SD or median ± quartile from at least three independent experiments. Variables for the different groups were compared using analysis of variance, the Mann–Whitney *U*-test and the chi-square test as appropriate. To determine the best cut-offs of continuous variables for predicting patient survival and pathologic response, the receiver operating characteristic (ROC) curve method was performed as described in a previous study^47^. A logistic regression model was used in the univariate and multivariate analyses. For internal validation, the models were subjected to 1000 bootstrap resamples. Survival curves were plotted by the Kaplan–Meier method, and differences were examined by the log-rank test. Statistical analyses were performed with SPSS 17.0 (SPSS Inc., Chicago, IL). *P* < 0.05 was considered statistically significant.

Reagents, materials and details relating to tissue IHC, immunofluorescence, RNA extraction, real-time PCR quantitation, western blotting analysis and ELISA are described in the Supplementary Materials and Methods. All primers used in real-time PCR are listed in Supplementary Table S3.

## Supplementary information

Supplementary Materials and Methods

Supplementary Figure S1

Supplementary Table S1

Supplementary Table S2

Supplementary Table S3

Supplementary figure legends
