# Supplementary material for: Cancer-associated fibroblasts promote cisplatin resistance in bladder cancer cells by increasing IGF-1/ERβ/Bcl-2 signalling
Source: Cell Death Dis. 2019 May 10;10(5):375. doi: 10.1038/s41419-019-1581-6 (PMC6510780; doi:10.1038/s41419-019-1581-6)
Supplement: Supplementary file 1 — Supplementary Materials and Methods [file 41419_2019_1581_MOESM1_ESM.docx]

**Supplementary Materials and Methods**

**Reagents and materials**

Anti-GAPDH antibody was purchased from Santa Cruz Biotechnology (CA, USA) and anti-ERβ, anti-Bcl-2, anti-phospho-Akt, anti-α-SMA, anti-cleaved caspase-3, anti-caspase-3 and anti-E-cadherin antibodies were obtained from ImmunoWay Biotechnology (catalogue numbers YT1637, YT0469, YP0007, YT5797, YM3431, YT6113, and YT1454, respectively; Newark, DE, USA). Anti-c-Jun, anti-phospho-Jun, anti-AKT, and anti-vimentin antibodies were purchased from Abzoom Biolabs (catalogue numbers BM0609, AM1159, AM2058, and AM4331, respectively; Dallas, TX, USA). Anti-human IGF-1 antibody and recombinant human IGF-1 were purchased from PeproTech (catalogue numbers 500-P11 and 100-11; Rocky Hill, NJ). Anti-PI3K, anti-phospho-IGF-1R, anti-IGF-1R and PI3K/AKT inhibitor LY294002 were purchased from Cell Signaling Technology (catalogue numbers 4292, 3024, 3027, and 9901S, respectively; Boston, MA, USA). Anti-FAP antibody was purchased from Abcam (ab28244, Cambridge, MA, USA). Cisplatin was purchased from Selleck Chemicals (S1166b, Houston, USA). Polyvinylidene difluoride membrane (PVDF) was obtained from Thermo Fisher Scientific (Rochester, NY).

**Tissue immunohistochemistry (IHC)**

For IHC staining, samples from the xenografted tumours in mice and human BCa tissues were fixed in 4% neutral-buffered paraformaldehyde, embedded in paraffin and then sliced into 4–5 μm thick sections. Before the slides were incubated with the corresponding primary antibodies and biotinylated secondary antibodies (Vector, Burlingame, CA), they were deparaffinized, hydrated, and subjected to antigen retrieval. The VECTASTAIN ABC peroxidase system was used to visualize the antibody binding in these sections. The following antibodies (with their dilutions) were used: 1) anti–ERβ (PC10; Novocastra, Newcastle upon Tyne, United Kingdom), 1:50; 2) anti-human α-SMA (ImmunoWay, YT5797), 1:100; and 3) Bcl-2 (clone 124; DakoCytomation, Denmark), 1:200. Nonimmunized mouse IgG antibody (Vector Laboratories) served as a negative control.

ERβ and Bcl-2 IHC scores in BCa were assessed using the German Immunoreactive Score system; in summary, the score was calculated by multiplying the staining intensity score (0, negative; 1, weak; 2, moderate; 3, strong) by the score corresponding to the percentage of immunoreactive cells (0% = 0; 1–10% = 1; 11–50% = 2; 51–80% = 3; 81–100% = 4).

Computer-assisted image analysis was used to assess α-SMA IHC staining. In brief, a microscope equipped with a charge-coupled device colour camera (Olympus Corp., Tokyo, Japan) was used to view the sections at high-power magnification. α-SMA expression in the BCa stroma was analysed according to previous studies^1^, and the relative percentage of the α-SMA area to the selected field area was analysed with an imaging processor using MacSCOPE software (Mitani Corp., Fukui, Japan). Since muscle fibres strongly express α-SMA, we avoided the muscle layer when analysing α-SMA expression.

Two investigators (JHC and ZBS) who were blinded to the clinicopathological data independently assessed the immunoreactivity. In cases of discrepant results, the cases were discussed until an agreement was reached.

**Immunofluorescence**

Cells at the proper density were plated on coverslips in 24-well plates and cultured. Before the cells were blocked with 5% BSA (Bovine serum albumin) in PBS for 60 min, they were fixed with 4% paraformaldehyde for 15 min and washed with PBS. The cells were then incubated with the corresponding primary antibodies (anti-E-cadherin, ImmunoWay, YT1454, dilution 1:400; anti-vimentin, ProteinTech, 60330-1-Ig, dilution 1:100; anti-α-SMA, ImmunoWay, YT5797, dilution 1:100; anti-IGF-1, Abcam, ab223567, dilution 1:100; anti-ERβ, ImmunoWay, YT1637, dilution 1:100) overnight at 4 °C. Then, before treatment with fluorescein isothiocyanate- or phycoerythrin-conjugated secondary antibodies (Cell Signaling Technology, Beverly, MA, USA), the cells were washed with PBS. DAPI (BBI, D6584, Cambridge, MA, USA) was used for counterstaining nuclei. A fluorescence microscope (Olympus DP72, Japan) was used to capture the images.

Double-fluorescence staining of either α-SMA and ERβ or α-SMA and IGF-1 was performed on formalin-fixed, paraffin-embedded tissue sections from tumours obtained from human clinical samples or from xenografted BCa tumours in nude mice. The sections were incubated with each primary antibody for 1 hr at room temperature followed by washes with phosphate-buffered saline. The sections were then incubated with an anti-rat antibody conjugated with Alexa Fluor 488 or an anti-mouse antibody conjugated with Alexa Fluor 568 (Invitrogen, USA) in the dark for 1 hr at room temperature. After the cells were washed with PBS twice, the nuclei were stained with DAPI (BBI, D6584, Cambridge, MA, USA) at room temperature for 10 min. The slides were analysed using a fluorescence microscope (Olympus DP72, Japan).

**RNA extraction and quantitation by real-time PCR**

TRIzol reagent (Invitrogen, Grand Island, NY) was used to isolate total RNA. In brief, one μg of total RNA was reverse transcribed using superscript III transcriptase (Invitrogen, Grand Island, NY). A Bio-Rad CFX96 system with SYBR Green was used to conduct quantitative real-time PCR (RT-PCR) to determine the mRNA expression level of the target genes. β-Actin was used to normalize the expression levels of the genes of interest. (All primers used are listed in Supporting Information Table S1).

**Western blotting analysis**

Cells were lysed in RIPA buffer, and an 8–10% SDS/PAGE gel was used to separate 20 μg of proteins, which were then transferred onto PVDF membranes. The membranes were blocked and incubated with appropriate dilutions of the corresponding primary antibodies. After the membranes were incubated with appropriate horseradish peroxidase (HRP)-conjugated secondary antibodies, the protein bands on the membranes were visualized using an ECL system (Thermo Fisher Scientific, Rochester, NY).

**ELISA assay**

CM was collected from single cultures of T24 and 5637 BCa cells, co-cultures of T24 or 5637 cells with CAFs and co-cultures treated with different concentrations of cisplatin for 48 hr with short hairpin RNA for the scramble sequence. Following the manufacturer's instructions, human IGF-1 ELISA kits (R&D Systems) were used to detect the concentration of IGF-1 in the CM.

**Identification and characterization of primary human CAFs and NFs**

CAFs and NFs were isolated from bladder tumour tissues and adjacent normal bladder mucosa of five patients. The isolated CAFs and NFs in culture displayed a thin and spindle-like morphology (Supporting Information Figure S1A and B), which indicated that the cells were indeed fibroblasts. To test the purity of the CAFs and NFs, we examined the expression of CAF-specific genes. Supporting Information Figure S1C shows the CAFs exhibited higher levels of FAP, FSP1, and ACTA2 (α-SMA) mRNA expression than did NFs, T24 cells or 5637 cells. Furthermore, the expression of several biomarkers, including vimentin (a mesenchymal cell marker), E-cadherin (an epithelial cell marker) and α-SMA (a myofibroblast marker), in these cells were tested by Western blotting. Strong E-cadherin expression was detected in T24 and 5637 BCa cells, vimentin was strongly expressed in both CAFs and NFs, and α-SMA was highly expressed in CAFs (Supporting Information Figure S1D and E). Western blotting and immunofluorescence confirmed that primary cultured fibroblast cells (CAFs and NFs) strongly expressed vimentin but only slightly expressed E-cadherin (Supporting Information Figure S1D and E). α-SMA expression was higher in CAFs than in NFs, T24 cells and 5637 cells (Supporting Information Figure S1D and E). Finally, the MTT assay was implemented to test the sensitivity of the above isolated NFs and CAFs to cisplatin, and the results show that compared with BCa cells, the isolated fibroblasts could endure a relatively wider range of cisplatin concentrations (Supporting Information Figure S1F). All of the above data suggest that we successfully isolated CAFs and NFs from the clinical specimens with high purity.

References

1. Hayashi, N. et al. Differential expression of cyclooxygenase-2 (COX-2) in epithelial cells and bile duct neoplasm. Hepatology 34, 638-650 (2001)
