# Supplementary figures and images for: Cancer-associated fibroblasts promote cisplatin resistance in bladder cancer cells by increasing IGF-1/ERβ/Bcl-2 signalling
Source: Cell Death Dis. 2019 May 10;10(5):375. doi: 10.1038/s41419-019-1581-6 (PMC6510780; doi:10.1038/s41419-019-1581-6)

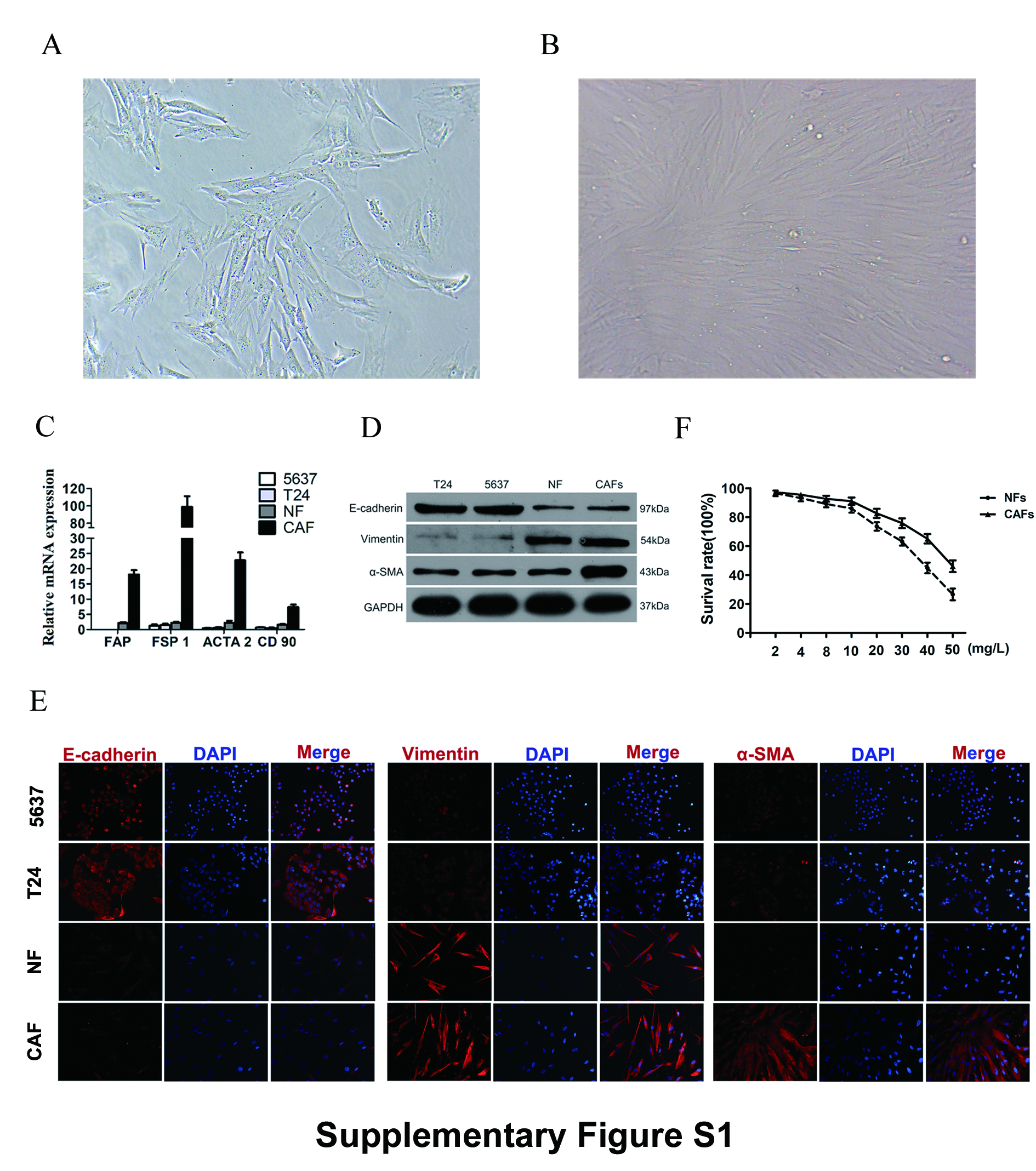

Supplement: Supplementary file 2 — Supplementary Figure S1 [file 41419_2019_1581_MOESM2_ESM.tif]
