# Supplementary material for: Cancer-associated fibroblasts promote cisplatin resistance in bladder cancer cells by increasing IGF-1/ERβ/Bcl-2 signalling
Source: Cell Death Dis. 2019 May 10;10(5):375. doi: 10.1038/s41419-019-1581-6 (PMC6510780; doi:10.1038/s41419-019-1581-6)
Supplement: Supplementary file 3 — Supplementary Table S1 [file 41419_2019_1581_MOESM3_ESM.docx]

| **Supplementary Table S1.**  The clinicopathological characteristics and pathologic outcomes of the patients. | | | | |  |  |  |
| --- | --- | --- | --- | --- | --- | --- | --- |
|  | | Total(n=28) | Complete pathologic response(n=7) | Pathologic partial response(n=12) | | Pathologic non-response(n=9) | Pv |
| Age,yr, (mean ± SD)^‡^ | | 60.9±6.6 | 58.6±6.3 | 61.6±7.9 | | 60.9±5.5 | 0.83 |
| BMI(kg/m2, mean ± SD)^‡^ | | 22.2±1.3 | 22.2±1.6 | 22.2±1.3 | | 23.2±1.3 | 0.97 |
| Male, n (%)^†^ | | 20(71.4) | 4(57.1) | 10(83.3) | | 6(66.6) | 0.43 |
| Clinical T stage, n (%)§ | |  |  |  | |  | 0.03^*^ |
| T2 | | 16(57.4) | 6(85.7) | 8(66.6) | | 2(22.2) |  |
| T3 | | 7(25.0) | 0(0.0) | 3(25.0) | | 4(44.4) |  |
| T4a | | 5(17.8) | 1(14.2) | 1(8.3) | | 3(33.3) |  |
| Grade,n (%)^†^ | |  |  |  | |  | 0.54 |
| Grade 1,2 | | 17(60.7) | 3(42.9) | 8(66.6) | | 6(66.6) |  |
| Grade 3 | | 11(39.3) | 4(57.1) | 4(33.3) | | 3(33.3) |  |
| Primary pathology TURBT, n (%)^†^ | |  |  |  | |  | 0.66 |
| Urothelial cancer | | 24(85.7) | 6(85.7) | 11(91.7) | | 7(77.8) |  |
| Urothelial cancer with squamous differentiation | | 2(7.1) | 1(14.3) | 0(0.0) | | 1(11.1) |  |
| Urothelial cancer with glandular differentiation | | 2(7.1) | 0(0.0) | 1(8.3) | | 1(11.1) |  |
| Associated CIS, n (%)^†^ | |  |  |  | |  | 0.91 |
| Yes | | 5（17.9） | 1（14.3） | 2（16.7） | | 2（25.6） |  |
| No | | 23（82.1） | 6（85.7） | 10（83.3） | | 7（77.8） |  |
|  |  |  |  |  |  |  |  |
| NAC Cycles n (%)^†^ | |  |  |  | |  | 0.13 |
| 3 | | 19（67.9） | 5（71.4） | 8（66.7） | | 6（66.7） |  |
| 4 | | 7（25.0） | 1（14.3） | 3（25.0） | | 3（33.3） |  |
| >4 | | 2（7.1） | 1（14.3） | 1（8.3） | | 0（0.0） |  |
| Extent of LND, n (%)^†^ | |  |  |  | |  | 0.33 |
| Standard | | 22（78.6） | 5（71.4） | 11（91.7） | | 6（66.7） |  |
| Extended | | 6（27.3） | 2（28.6） | 1（8.3） | | 3（33.3） |  |
| Smoking history, n (%)^†^ | | 19（67.9） | 5（71.4） | 7（58.3） | | 7（77.8） | 0.62 |

*：P<0.05

†：Chi-square test, partitions of the Chi-square method was used for multiple comparison between groups.

‡：Analysis of variance (ANOVA) with equal variances assumed under Levene's test

§: Mann-Whitney U test
