# Supplementary material for: Cancer-associated fibroblasts promote cisplatin resistance in bladder cancer cells by increasing IGF-1/ERβ/Bcl-2 signalling
Source: Cell Death Dis. 2019 May 10;10(5):375. doi: 10.1038/s41419-019-1581-6 (PMC6510780; doi:10.1038/s41419-019-1581-6)
Supplement: Supplementary file 4 — Supplementary Table S2 [file 41419_2019_1581_MOESM4_ESM.docx]

**Supplementary Table S2**

| Univariate and multivariable analysis of factors predicting a pathologic non-response (ypT ≥ypT2) | | | | | | | |
| --- | --- | --- | --- | --- | --- | --- | --- |
| Variable | Univariate | | | Multivariate | | | |
|  | HR^‡^ | 95%CI^†^ | P | HR | 95%CI | | P |
| Primary pathology TURBT |  |  |  |  |  | |  |
| UC with squamous differentiation | 1.14 | 0.88-14.77 | 0.92 | - | - | | - |
| UC with glandular differentiation | 2.29 | 0.12-41.9 | 0.58 | - | - | | - |
| UC（ref） |  |  |  |  |  | |  |
| Associated CIS |  |  |  |  |  | |  |
| Yes | 1.52 | 0.21-11.23 | 0.68 | - | - | | - |
| No（ref） |  |  |  |  |  | |  |
| NAC Cycles |  |  |  |  |  | |  |
| 4 or more | 1.08 | 0.20-5.87 | 0.93 | - | - | | - |
| 3（ref） |  |  |  |  |  | |  |
| Grade |  |  |  |  |  | |  |
| Grade 3 | 1.45 | 0.28-7.63 | 0.66 | - | - | | - |
| Grade 1,2（ref） |  |  |  |  |  | |  |
| Clinical T stage |  |  |  |  |  |  | |
| ≥T3 | 9.80 | 1.50-63.85 | 0.017^*^ | 21.88 | 1.22-393.61 | | 0.036^*^ |
| ≤T2（ref） |  |  |  |  |  | |  |
| Gender |  |  |  |  |  | |  |
| Male | 1.40 | 0.25-7.83 | 0.70 | - | - | | - |
| Female（ref） |  |  |  |  |  | |  |
| Age(continuous) | 1.00 | 0.89-1.13 | 1.00 | - | - | | - |
| Biopsy ERβ scores ERβ scores |  |  |  |  |  | |  |
| High-level ERβ scores>3.5 | 13.12 | 1.92-89.52 | 0.009^*^ | 41.26 | 1.84-958.78 | | 0.019^*^ |
| Low-level ERβ scores<3.5（ref） |  |  |  |  |  | |  |
| Biopsy α-SMA scores |  |  |  |  |  | |  |
| High-level α-SMA scores>7.85 | 7.50 | 1.28-44.09 | 0.026^*^ | 10.95 | 0.65-185.13 | | 0.097 |
| Low-level α-SMA scores<7.85（ref） | |  |  |  |  | |  |

‡ HR: hazard ratio.

†95% CI: 95% confidence interval.

*Statistically significant at P<0.05.
