# Supplementary material for: Cancer-associated fibroblasts promote cisplatin resistance in bladder cancer cells by increasing IGF-1/ERβ/Bcl-2 signalling
Source: Cell Death Dis. 2019 May 10;10(5):375. doi: 10.1038/s41419-019-1581-6 (PMC6510780; doi:10.1038/s41419-019-1581-6)
Supplement: Supplementary file 5 — Supplementary Table S3 [file 41419_2019_1581_MOESM5_ESM.doc]

| **Supplementary Table S3**: Primers for q-PCR | | |
| --- | --- | --- |
| Target genes | Sense | Antisense |
| ERβ | AGCACGGCTCCATATACATACC | TGGACCACTAAAGGAGAAAGGT |
| IGF-I | GCTCTTCAGTTCGTGTGTGG | TGACTTGGCAGGCTTGAGG |
| FAP | ATGAGCTTCCTCGTCCAATTCA | AGACCACCAGAGAGCATATTTTG |
| ACTA2 | GTGTTGCCCCTGAAGAGCAT | GCTGGGACATTGAAAGTCTCA |
| FSP1 | GATGAGCAACTTGGACAGCAA | CTGGGCTGCTTATCTGGGAAG |
| CD90 | ATCGCTCTCCTGCTAACAGTC | CTCGTACTGGATGGGTGAACT |
| BCL-2  Bax  Bcl-x  Bak  Bcl-W  MCL-1  Bad  Survivin | CAACATCACAGAGGAAGTAGA  TTTGCTTCAGGGTTTCATCCA  GAACAGGATACTTTTGTGGAACT  TGCCACCAGCCTGTTTGA  TCACCCTACCCTCTACCACA  TAAGGACAAAACGGGACTGG  TGTGGACTCCTTTAAGAAGGGAC  GAACTGGCCCTTCTTGGAG | CACTTGATTCTGGTGTTTC  CGGCGGCAATCATCCTCT  GGTGGGAGGGTAGAGTGGA  CAGTGATGCAGCATGAAGTCG  AGACCAAGCCCTTTACCCT  ACCAGCTCCTACTCCAGCAA  GGGCAGTGGGAACGGGTG  AAGTCTGGCTCGTTCTCAGTG |
| CHIP-ERβ | ACTGGCTCCTTAGAATCAGACAT | ATTTAAGAGGTCTGGAGTAGGGC |
| β-actin | CATGTACGTTGCTATCCAGGC | CTCCTTAATGTCACGCACGA |
