# Supplementary material for: Cancer-associated fibroblasts promote cisplatin resistance in bladder cancer cells by increasing IGF-1/ERβ/Bcl-2 signalling
Source: Cell Death Dis. 2019 May 10;10(5):375. doi: 10.1038/s41419-019-1581-6 (PMC6510780; doi:10.1038/s41419-019-1581-6)
Supplement: Supplementary file 6 — Supplementary figure legends [file 41419_2019_1581_MOESM6_ESM.docx]

**FIGURE LEGENDS**

**Figure 1**: The clinicopathological data of 28 MIBC patients who received cisplatin-based NAC. A, B: The expression of α-SMA (A) and ERβ (B) in adjacent normal bladder, pretreatment biopsy and resected RC samples (α-SMA×200, ERβ×100). C: α-SMA (left) and ERβ (right) scores in the adjacent normal bladder with or without NAC treatment and in the pretreatment biopsy and resected RC specimens with different pathological outcomes. D: The correlation of α-SMA expression in the cancer-associated stroma and BCa ERβ expression in the pretreatment biopsy tissues (left) and RC (right) tissues. E, F: Kaplan-Meier survival curves of OS (E) and PFS (F) in the 28 patients. The results indicated that α-SMA and ERβ expression was related to OS and PFS. Data are presented as the mean ± SD. *P < 0.05.

**Figure 2**: BCa cells can better promote the transformation of NFs into CAFs than non-malignant bladder cells, and appropriate concentrations of cisplatin can accelerate this process. A: Cartoon showing the transwell co-culture system: CM from BCa cells or SVHUCs was plated into the lower chambers of the transwells, and 1 × 10^5^ NFs cells were plated onto the upper chambers. When implementing fibroblast recruitment assays, a polycarbonate membrane with a 5-μm pore size was inserted between the two chambers. For the noncontact co-culture system, a polycarbonate membrane with a 0.4-μm pore size was used. B: After 8 hr of incubation, the fibroblasts that migrated into the lower chambers were collected and counted. C: The α-SMA immunofluorescence staining assay was implemented on all fibroblasts in the noncontact co-culture system after 8 hr of incubation, and the percentage of α-SMA(+) fibroblasts was counted. D: Western blot and q-PCR results showed the expression of the CAF-specific markers ACTA2 (α-SMA) and FAP in fibroblasts in the noncontact co-culture system after 8 hr of incubation. E: Different concentrations of cisplatin were added to the noncontact co-culture system. After 8 hr of incubation, the protein and mRNA levels of ACTA2 and FAP in fibroblasts were analysed. F, G: Different concentrations of cisplatin were added to the noncontact co-culture system. After 8 hr of incubation, the percentage of α-SMA(+) fibroblasts was counted in the upper chambers using immunofluorescence. The first column of Fig. 2G (no cisplatin added) is also the experimental condition of Fig. 2C. In the q-PCR analysis, we used the β-actin gene as the normalization control. Data are presented as the mean ± SD. *P < 0.05.

**Figure 3**: CAFs could reduce BCa cell sensitivity to cisplatin, promote BCa cell proliferation and decrease cisplatin-induced apoptosis. A: The cell viability with different concentrations of cisplatin as measured by the MTT assay. B: The IC50 values in each group. C: Colony formation assay showed that after 2 weeks of culture, compared with the non-co-culture groups, the co-cultured T24 and 5637 cell groups showed increased colony forming ability. All groups were treated with a specific concentration of cisplatin (T24, 25 mg/L and 5637, 6 mg/L). D and E: Apoptotic cells were detected by Annexin V-FITC and PI double staining and analysed by flow cytometry. The result showed that co-culturing reduced cisplatin-induced apoptosis (T24, 25 mg/L and 5637, 6 mg/L respectively). F: Total and cleaved caspase-3 levels in BCa cells were determined by Western blotting for each group. T24 and 5637 cells grown with (+) or without (-) CAF co-culture (24 h) were incubated for 24 hr in the presence (+) or absence (-) of 25 mg/L and 6 mg/L cisplatin, respectively. The histogram on the right illustrates the ratio of cleaved caspase-3 and total caspase-3. Data are presented as the mean ± SD. *P < 0.05.

**Figure 4**: CAFs could decrease BCa apoptosis induced by cisplatin via upregulation of ERβ/Bcl-2 signalling. A: q-PCR results showed increased ERβ expression in BCa cells (T24 and 5637) after co-culture with CAFs. The mean expression value of the control cells (T24, 5637) was defined as 1. B: Western blotting results showed that ERβ protein expression increased in BCa cells (T24, 5637) after co-culture with CAFs. C: Validation of the ERβ shRNA knockdown efficiency in T24 and 5637 cells using Western blotting (right) and relative mRNA expression (left) levels of ERβ in T24 and 5637 cells at 48 hr after transfection. Expression values were shown relative to those of the untreated group (control=100%) (left). D, E: Knocking down ERβ in BCa cells can reverse the effects of CAFs on BCa cell resistance to apoptosis induced by cisplatin (T24, 25 mg/L; 5637, 6 mg/L). F, qRT-PCR results showed that Bcl-2 expression levels were significantly increased in both T24 and 5637 cells after co-culture with CAFs and could be partially reversed by ERβ knockdown. The mean expression value of the control cells (T24, 5637) was defined as 1. G: Western blotting results showed that Bcl-2 expression was increased in T24 and 5637 cells after co-culture with CAFs and decreased after knocking down ERβ. All procedures were conducted after cells were cultured in an appropriate concentration of cisplatin (T24, 25 mg/L; 5637, 6 mg/L). Data are presented as the mean ± SD. *P < 0.05.

**Figure 5**: Elucidation of the mechanism by which CAFs increased ERβ expression in BCa cells. A: IGF-1 levels in the CM from CAFs, BCa cells, or co-cultured (CAF+BCa) cells were quantified by ELISA (left panel). IGF-1 levels in the CM of co-cultured cells treated with different concentrations of cisplatin for 48 hr were quantified by ELISA (right panel). B: Assessment of IGF-1 mRNA expression in CAFs by qRT-PCR using the β-actin gene as the normalization control. C: Immunoﬂuorescence double staining for α-SMA (green) and IGF-1 (red) in pathologic non-response RC tissues (400×). Double-positive areas are indicated (arrows). D: Western blotting (left panel) and qRT-PCR (right panel) results showed that blocking IGF-1 with an anti-IGF-1 neutralizing antibody can partially reverse CAF-mediated ERβ and Bcl-2 upregulation in BCa cells. The mean expression value of the control cells (T24, 5637) was defined as 1. E: Blocking IGF-1 can partially reverse the effects of CAFs on BCa cell resistance to apoptosis induced by cisplatin. F: Western blotting (left) and immunofluorescence staining (right) show that blocking IGF-1 in the co-culture system reversed the capacity of BCa cells to induce NF transformation into CAFs (The expression of α-SMA in the fibroblasts decreased after blocking IGF-1 in the co-culture system). G: Blocking IGF-1 in the co-culture system reversed the capacity of the BCa to recruit fibroblasts. H: IGF-1 increased the protein (left panel) and mRNA (right panel) levels of ERβ and Bcl-2 in BCa cells. I: IGF-1 can increase BCa cell resistance to cisplatin. The procedures in D, E, H, and I were conducted after the cells were incubated in an appropriate concentration of cisplatin (T24, 25 mg/L; 5637, 6 mg/L). Data are presented as the mean ± SD. *P < 0.05.

**Figure 6**: CAFs increased ERβ expression via IGF-1/AKT/c-Jun signalling. A: The expression of IGF-1R and PI3K and the phosphorylation of the IGF-1R and AKT proteins were increased after co-culture, but these effects were reversed by blocking IGF-1. B: Western blotting showed that adding the IGF-1R inhibitor AG (1 μM) to the co-culture system could partially reverse CAF-mediated increases in the protein expression of ERβ and Bcl-2 and the phosphorylation of IGF-1R and AKT. C: Western blotting showed that ERβ and Bcl-2 expression and AKT phosphorylation were decreased when the cells were pretreated with the AKT inhibitor LY (10 μM) in the co-culture system. D: qRT-PCR results showed changes in ERβ and Bcl-2 mRNA expression in BCa cells after co-culture upon pretreatment with AG (1 μM) and LY (10 μM) for 1 hr before co-culture. The mean expression value of the control cells (T24, 5637) was defined as 1.0. E: Both AG and LY can partially reverse the effects of CAFs on BCa cell resistance to cisplatin-induced apoptosis. F: Phosphorylation of c-Jun was detected by Western blotting in each group. G: The cells were treated with or without IGF-1 for 8 hr. Then, the cells were harvested and subjected to chromatin ChIP with anti-c-Jun or control IgG, followed by q-PCR. H: ChIP products were measured by real-time PCR. The expression value in the control group was defined as 1. All procedures were conducted after cells were cultured in an appropriate concentration of cisplatin (T24, 25 mg/L; 5637, 6 mg/L). Data are presented as the mean ± SD. *P < 0.05.

**Figure 7**: CAFs reduced BCa sensitivity to cisplatin by stimulating IGF-1/ERβ/Bcl-2 signalling in vivo. A: Grouping and intervention schemes. B: Changes in the tumour volumes of each group in the verification and intervention study. Tumour volumes were calculated as described in the Materials and Methods. Arrows represent the start of cisplatin treatment, n=5 per group. The mice were sacrificed after completion of the treatments. C, D: Quantification of the tumour sizes in each group in the verification and intervention studies after mice were sacrificed. E: The percent reduction of tumour burden was calculated to evaluate the effect of cisplatin treatment as follows: percent reduction of tumour burden= (no treatment group – treatment group)/ no treatment group×100%. F: IHC staining for α-SMA, ERβ and Bcl-2 in xenograft bladder tumour tissues (α-SMA, 200×; ERβ and Bcl-2, 100×) in each group. G: Double fluorescence staining of α-SMA (green) and IGF-1 (red) in xenograft bladder tumour tissues (400×). Data are presented as the mean ± SD. *P < 0.05.

**Figure 8**: Cartoon illustration summarizing CAF-enhanced cisplatin resistance via stimulating IGF-1/ERβ/Bcl-2 signalling in the tumour microenvironment of BCa. The red arrows indicated an increase in ERβ and Bcl-2 expression.

**Supplementary Figure S1**: Identification and characterization of primary human NFs and CAFs. A, B: Isolated CAFs (A) and NFs (B) were photographed on a brightfield microscope (magnification 200×). C: The mRNA expression levels of CAF-specific genes, including FAP, FSP1 and ACTA2, were assessed in 5637 cells, T24 cells (a control epithelial cell line), NFs and CAFs by qRT-PCR using β-actin as the normalization control. D: Western blotting assay showed the protein expression levels of Vimentin, E-cadherin and α-SMA in 5637 cells, T24 cells, NFs and CAFs. E: The subcellular location and expression of E-cadherin, Vimentin and α-SMA in 5637 cells, NFs and CAFs were detected by immunofluorescence. F: The viability of NFs and CAFs viability in the presence of different concentrations of cisplatin was measured by the MTT assay. Data are presented as the mean ± SD.
